# *Arntl* deficiency in myeloid cells reduces neutrophil recruitment and delays skeletal muscle repair

**DOI:** 10.1038/s41598-023-33830-8

**Published:** 2023-04-25

**Authors:** Aiko Watanabe, Hiroyuki Koike, Naoki Kumagami, Shigeki Shimba, Ichiro Manabe, Yumiko Oishi

**Affiliations:** 1 Department of Biochemistry and Molecular Biology, Nippon Medical School, 1-1-5 Sendagi, Bunkyo-ku, Tokyo, 113-8602 Japan; 2 Department of Molecular Cell Biology, Graduate School of Medical and Dental Sciences, Tokyo Medical and Dental University, 1-5-45, Yushima, Bunkyo-ku, Tokyo, 113-8510 Japan; 3 Department of Health Science, School of Pharmacy, Nihon University, 7-7-1 Narashinodai, Funabashi, Chiba, 274-8555 Japan; 4 Department of Systems Medicine, Graduate School of Medicine, Chiba University, 1-8-1 Inohana, Chuo-ku, Chiba-shi, Chiba, 260-8670 Japan

**Keywords:** Biochemistry, Immunology, Cell biology

## Abstract

After a muscle injury, a process comprising inflammation, repair, and regeneration must occur in a time-sensitive manner for skeletal muscle to be adequately repaired and regenerated. This complex process is assumed to be controlled by various myeloid cell types, including monocytes and macrophages, though the mechanism is not fully understood. Aryl hydrocarbon receptor nuclear translocator-like (*Arntl* or *Bmal1*) is a transcription factor that controls the circadian rhythm and has been implicated in regulating myeloid cell functions. In the present study, we generated myeloid cell-specific *Arntl* conditional knockout (cKO) mice to assess the role of *Arntl* expressed in myeloid cell populations during the repair process after muscle injury. Myeloid cell-specific *Arntl* deletion impaired muscle regeneration after cardiotoxin injection. Flow cytometric analyses revealed that, in cKO mice, the numbers of infiltrating neutrophils and Ly6C^hi^ monocytes within the injured site were reduced on days 1 and 2, respectively, after muscle injury. Moreover, neutrophil migration and the numbers of circulating monocytes were significantly reduced in cKO mice, which suggests these effects may account, at least in part, for the impaired regeneration. These findings suggest that *Arntl,* expressed in the myeloid lineage regulates neutrophil and monocyte recruitment and is therefore required for skeletal muscle regeneration.

## Introduction

Skeletal muscle has a remarkable capacity to regenerate after acute injury or damage. During the regeneration process, satellite cells, which are tissue stem cells, proliferate, differentiate, and fuse to replace damaged fibers at the injury site. This regeneration process as well as the associated inflammatory response are tightly orchestrated, and the coordinated temporal interaction between satellite and myeloid cells plays an important role^1,2^. In models of disrupted recruitment of myeloid cells, such as chemokine (C–C motif) receptor 2 (*Ccr2*) deficient^3–6^, chemokine (C-X3-C motif) receptor 1 (*Cx3cr1*) deficient^7^, and CD11b^+^ cell-depleted^8^ mice, there are significant defects in the repair of injured skeletal muscle. During the early regeneration stage, neutrophils and Ly6C^hi^ inflammatory monocytes/macrophages infiltrate the injured area^3^, where they induce satellite cell activation and proliferation^8^. At least some of these Ly6C^hi^ inflammatory monocytes/macrophages become reparative macrophages in the repair phase^1^. Anti-inflammatory macrophages predominate during the repair phase and produce cytokines that promote myogenic differentiation, fusion, and maturation^8–10^. These findings suggest that mechanisms that regulate time-dependent changes in myeloid cell function are critical for muscle regeneration.

Aryl hydrocarbon receptor nuclear translocator-like (*Arntl*, also known as *Bmal1*) is a transcription factor that regulates gene expression related to myeloid cell function and is a clock gene that controls circadian rhythms. *Arntl*-deficient macrophages exhibit an exaggerated inflammatory response and secrete high levels of pro-inflammatory cytokines, including IL-6^11–14^. We previously reported that macrophage function changes during the inflammatory process and that ARNTL is responsible for that time-dependent change of macrophage function^15^. This implies that the loss of *Arntl* would profoundly affect the regulation of myeloid cell function. In addition, under some conditions, such as the unique environment of space, *Arntl* expression is altered and circadian rhythms may be disrupted in peripheral tissues, including skeletal muscle^16,17^. Under such conditions, the peripheral clock of myeloid cells may also be disrupted, affecting regeneration and repair after tissue damage. However, it is still unknown how ARNTL-mediated modulation of the peripheral clock of myeloid cells affects the skeletal muscle tissue regeneration process.

Mice in which *Arntl* has been systemically deleted exhibit a significant delay in skeletal muscle regeneration and diminished exercise capacity accompanied by muscle loss^18–20^. However, following skeletal muscle-selective deletion of *Arntl*, mice exhibit normal locomotor activity, and their skeletal muscle exhibits no sign of abnormal phenotypes^21–23^. Because skeletal muscle regeneration requires myeloid cells, we hypothesized that the mechanisms controlled by *Arntl* in myeloid cells are essential for proper muscle regeneration. To test that idea, in the present study, we induced muscle injury in myeloid cell-specific *Arntl*-deficient mice and analyzed their muscle regeneration over time to determine how skeletal muscle regeneration is affected in an environment where the macrophage clock is disrupted.

## Results

### *Arntl* expression in myeloid cells is essential for muscle regeneration

**Figure 1:**
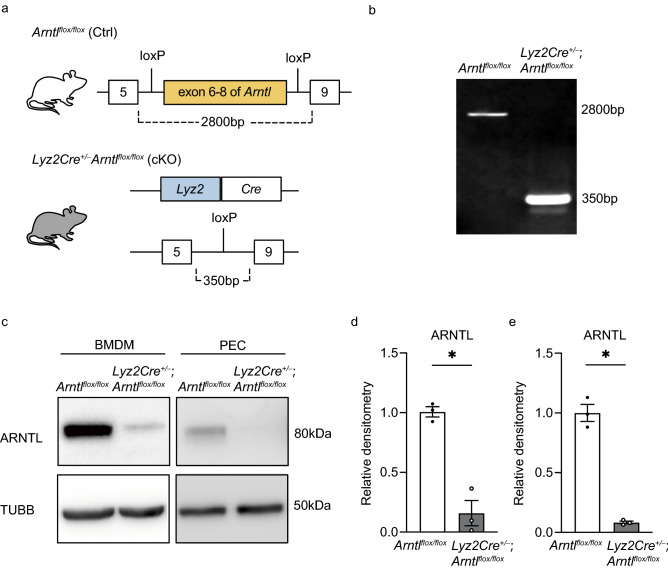
Generation of the myeloid cell-specific *Arntl* knockout model. (**a**) Schematic representation of the gene targeting method used to generate the myeloid cell-specific aryl hydrocarbon receptor nuclear translocator-like (*Arntl*) knockout mice. (**b**) *Arntl* genotyping. PCR of the floxed (wild-type) allele produced a 2800-bp product from control (Ctrl) bone marrow-derived macrophages (BMDM) DNA, whereas PCR of the deleted allele produced a 350-bp product from DNA in the conditional knockout (cKO) BMDM. Raw gel image is in Fig. S7. (**c**) Western blots of ARNTL and tubulin beta (TUBB) in BMDM and peritoneal exudate cells (PEC) from Ctrl and cKO mice. The grouping images of ARNTL and TUBB from different parts and exposures of the same membrane. Raw blot images are in Fig. S8. The results are representative of three independent experiments. (**d**,**e**); Densitometry of BMDM (**d**) and PEC (**e**) western blots from three independent experiments. Data are expressed as the means ± SEM. * *p* < 0.05 with unpaired two-tailed Student’s *t*-test.

To test whether *Arntl* expression in macrophages is required for skeletal muscle regeneration, we crossed a mouse carrying floxed exons 6–8 in the *Arntl* gene locus with the *Lyz2Cre* line^24^, which expresses Cre-recombinase in their myeloid lineage^25^ (Fig. 1a,b). Western blotting using bone marrow-derived macrophages and peritoneal exudate cells confirmed *Arntl* deletion in *Lyz2Cre*^+*/−*^* Arntl *^*flox/flox*^ (cKO) mice (Fig. 1c–e). Although systemically *Arntl*-deficient mice show a decrease in skeletal muscle weight per total body weight despite a decrease in total body weight^19^, deletion of *Arntl* from myeloid cells did not affect body weight (Fig. S1a), TA muscle weight per body weight (Fig. S1b), or skeletal muscle fiber diameters (Fig. S1c–f).

**Figure 2:**
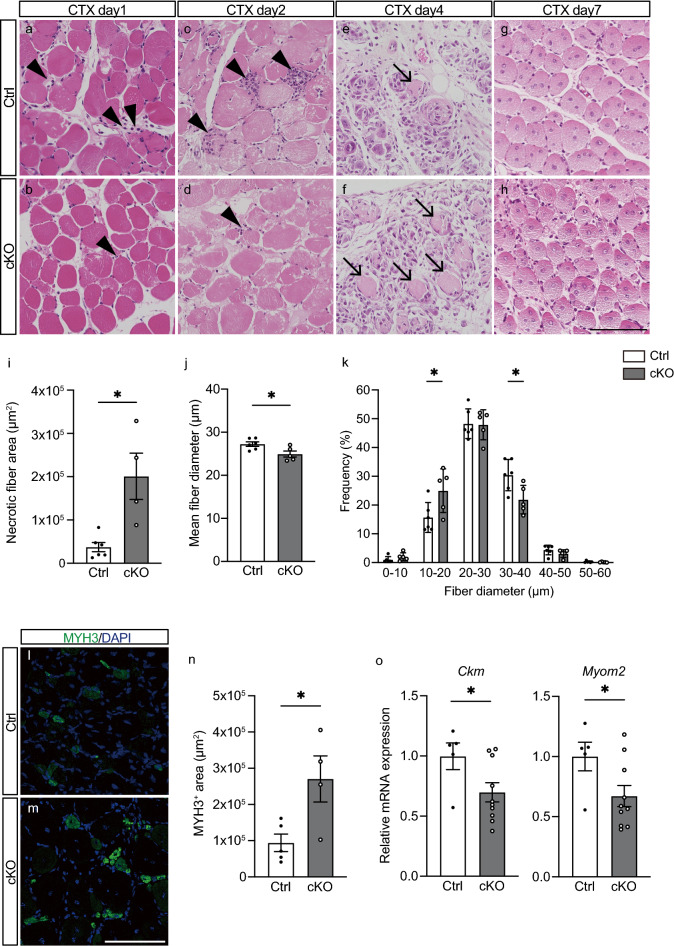
*Arntl* deletion from myeloid cells induces abnormal muscle regeneration. (**a**–**h**) Hematoxylin/eosin staining of Ctrl and cKO tibialis anterior (TA) muscles on days 1 (**a**, **b**), 2 (**c**, **d**), 4 (**e**, **f**), and 7 (**g**, **h**) after cardiotoxin (CTX) injury. Arrowheads indicate infiltration of inflammatory cells into the muscle on days 1 and 2 post-injury. Arrows indicate necrotic myofibers in the muscle 4 days post-injury. Representative images from Ctrl (n = 4) and cKO (n = 4) muscles on day 1, Ctrl (n = 4) and cKO (n = 4) muscles on day 2, Ctrl (n = 6) and cKO (n = 4) muscles on day 4, and Ctrl (n = 6) and cKO (n = 5) muscles on day 7. (**i**) Quantification of necrotic myofiber area of Ctrl (n = 6) and cKO (n = 5) TA muscles on day 4 post-injury. (**j**) Mean myofiber diameter in Ctrl (n = 6) and cKO (n = 5) TA muscles on day 7 post-injury. (**k**) Myofiber diameter distributions in Ctrl (n = 6) and cKO (n = 5) TA muscles on day 7 post-injury. (**l**, **m**) Immunostaining for myosin heavy polypeptide 3 (MYH3, green) and DAPI staining of nuclei (blue) in Ctrl (n = 5) and cKO (n = 4) TA muscles on day 7 post-injury. (**n)** Quantification of MYH3-positive area in Ctrl (n = 5) and cKO (n = 4) TA muscles on day 7 post-injury. (**o)** RT-PCR assessment of creatine kinase, muscle (*Ckm*) and myomesin 2 (*Myom2*) expression in TA muscles from Ctrl (n = 5) and cKO (n = 10) mice on day 7 post-injury. Data are expressed as the means ± SEM. * *p* < 0.05 with unpaired two-tailed Student’s *t*-test (**i**, **j**, **n**, **o**) or two-way ANOVA with Bonferroni’s multiple comparisons test (**k**). Scale bar = 100 μm.

We then induced skeletal muscle injury in Ctrl and cKO mice by injecting cardiotoxin (CTX) into the tibialis anterior (TA) muscle and performed histological analyses of the post-injury inflammatory and regenerative processes (Fig. 2a–h). Intramuscular injection of CTX causes rapid muscle degeneration followed by recruitment of inflammatory cells and muscle regeneration at the injury site^5,26^. On days 1 and 2 after injury, swollen necrotic fibers^2,4^ and infiltrating inflammatory cells were observed within the TA muscle in Ctrl mice (Fig. 2a,c). During the same period, cKO mice showed less inflammatory cell infiltration than Ctrl mice (Fig. 2b,d). By day 4 after injury in Ctrl mice, most parts of the necrotic fibers had been phagocytized by immune cells^27^, whereas a substantial numbers of necrotic myofibers remained in the cKO muscle 4 days after injury (Fig. 2e,f,i). By day 7, the phagocytized myofibers had been replaced by regenerating fibers with central nuclei in the Ctrl muscle (Fig. 2g). The number of regenerating muscle fibers with central nuclei tended to be higher in cKO fibers, but the increase did not reach the level of significance as compared to Ctrl (Fig. S2). The mean diameters of regenerating fibers were 9.3% smaller in cKO than Ctrl mice (Fig. 2h,j). Analysis of the distribution of myofiber diameters confirmed that fiber size was smaller in cKO than Ctrl mice (Fig. 2k). Immunofluorescent staining of TA muscles on day 7 after injury showed that the expression of MYH3, a marker of immature myofibers^28,29^, was higher in cKO than Ctrl mice (Fig. 2l–n). Additionally, expression levels of creatine kinase, muscle (*Ckm*) and myomesin 2 (*Myom2*), two markers of mature regenerating myofibers^30–32^, were lower in cKO than Ctrl mice on day 7 (Fig. 2o). These results suggest that regeneration is delayed in myeloid cell-specific *Arntl*-deficient mice.

It was previously suggested that *Arntl* deficiency leads to increased induction of inflammatory cytokines via TLR4 activation in macrophages^11–14^. To determine whether that effect was reproduced in our *Arntl* deficient macrophages, we analyzed cultured peritoneal macrophages stimulated with 100 ng/mL KLA. The observed higher mRNA and protein expression of *Il6* in cKO than Ctrl macrophages recapitulated the phenotype reported in earlier studies^13,14^ (Fig. S3a,b). We then isolated myeloid cells (CD45^+^ CD11b^+^) from the injured muscles of cKO and Ctrl mice on day 3 after injury and compared the *Il6* mRNA expression, and the expression was increased in the cKO myeloid cells (Fig. S3c). Previous studies found that IL6 produced by inflammatory monocytes and macrophages at an early phase of injury is essential for activating muscle stem cells and initiating the regenerative process^8,10,33^. In *Arntl*-cKO mice, therefore, the sustained IL6 production may have inhibited muscle regeneration. To test that hypothesis, we further investigated the crosstalk between macrophages and muscle stem cells in vitro. Peritoneal macrophages were obtained from cKO and Ctrl mice and cultured, after which the conditioned media were collected. Muscle stem cells collected from wild-type mice were then cultured with the conditioned media for 48 h. The results showed that muscle stem cell differentiation was decreased in the medium conditioned by cKO macrophages (Fig. S4), which suggests *Arntl* deficiency may alter the phenotype and function of macrophages.

### *Arntl* deletion in myeloid cells reduces neutrophil and monocyte recruitment into injured muscles

**Figure 3:**
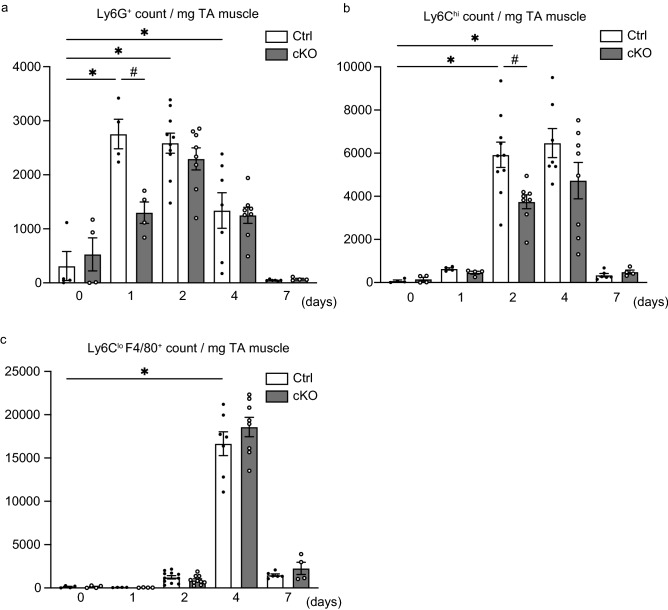
*Arntl* deletion from myeloid cells reduces the numbers of neutrophils and monocytes infiltrating injured muscle. (**a**) Quantification of neutrophils (CD45^+^ CD11b^+^ Ly6G^+^) in untreated Ctrl (n = 4) and cKO (n = 4) TA muscles and in Ctrl (n = 4) and cKO (n = 4) muscles on day 1, Ctrl (n = 10) and cKO (n = 8) muscles on day 2, Ctrl (n = 7) and cKO (n = 8) muscles on day 4, and Ctrl (n = 6) and cKO (n = 4) muscles on day 7 after CTX injury. (**b**) Quantification of monocytes (CD45^+^ CD11b^+^ Ly6G^-^ Ly6C^hi^) in untreated Ctrl (n = 4) and cKO (n = 4) TA muscles and in Ctrl (n = 4) and cKO (n = 4) muscles on day 1, Ctrl (n = 10) and cKO (n = 8) muscles on day 2, Ctrl (n = 7) and cKO (n = 8) muscles on day 4, and Ctrl (n = 6) and cKO (n = 4) muscles on day 7 after CTX injury. (**c**) Quantification of macrophages (CD45^+^ CD11b^+^ Ly6G^-^ Ly6C^lo^) in untreated Ctrl (n = 4) and cKO (n = 4) TA muscles and in Ctrl (n = 4) and cKO (n = 4) muscles on day 1, Ctrl (n = 10) and cKO (n = 8) muscles on day 2, Ctrl (n = 7) and cKO (n = 8) muscles on day 4, and Ctrl (n = 6) and cKO (n = 4) muscles on day 7 after CTX injury. Data are expressed as the means ± SEM. * *p* < 0.05 vs. untreated Ctrl muscles, # *p* < 0.05 vs. Ctrl muscles per day with two-way ANOVA with Bonferroni’s multiple comparisons test.

Given the histological result showing reduced inflammatory cell infiltration of the injured area in cKO mice, we next addressed which subpopulations of inflammatory cells were affected by the deletion of *Arntl* from myeloid cells. Flow cytometric analysis of the intramuscular myeloid cells within injured TA muscles revealed that, in Ctrl mice, the numbers of neutrophils (CD45^+^ CD11b^+^ Ly6G^+^) were markedly increased after injury, peaking 1 day post-injury, and that the numbers of infiltrating neutrophils were lower in cKO than Ctrl mice on day 1 (Fig. 3a). In Ctrl mice, numbers of monocytes (CD45^+^ CD11b^+^ Ly6G^-^ Ly6C^hi^) continued to increase on days 2 and 4, but had decreased significantly by day 7 (Fig. 3b). As with neutrophils, the numbers of monocytes infiltrating the injured TA muscle were lower in cKO than Ctrl mice (Fig. 3b). The number of Ly6C^lo^ macrophages (CD45^+^ CD11b^+^ Ly6G^-^ Ly6C^lo^) increased on day 4 in both Ctrl and cKO, but there were no significant differences between genotypes (Fig. 3c). Thus, *Arntl* deficiency in myeloid cells limited muscle recruitment of neutrophils and monocytes in response to acute injury.

### *Arntl* is needed for neutrophil chemotaxis during skeletal muscle injury

**Figure 4:**
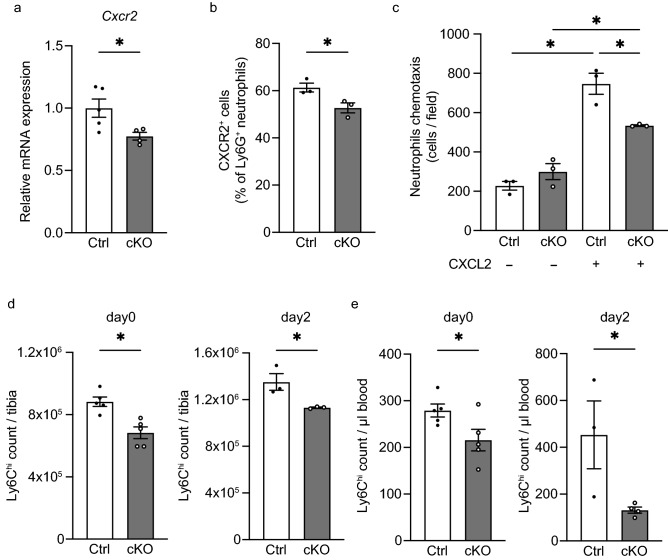
*Arntl* deletion from myeloid cells decreases expression of *Cxcr2* related to neutrophil migration and reduces monocyte numbers in bone marrow and peripheral blood. (**a**) RT-PCR assessment of chemokine (C-X-C motif) receptor 2 (*Cxcr2*) expression in neutrophils (CD45^+^ CD11b^+^ Ly6G^+^) sorted from untreated bone marrow cells from Ctrl (n = 5) and cKO (n = 4) mice. (**b**) Percentage of CXCR2^+^ cells among Ctrl (n = 3) and cKO (n = 3) bone marrow CD45^+^ CD11b^+^ Ly6G^+^ neutrophils. (**c**) Numbers per field of migrated Ctrl (n = 3) and cKO (n = 3) neutrophils left untreated or stimulated with 100 ng/ml CXCL2. (**d)** Quantification of monocytes (CD45^+^ CD11b^+^ Ly6G^-^ Siglec-F^-^ Ly6C^hi^) among control (Ctrl) and cKO bone marrow cells from mice left untreated (n = 5) and on day 2 after muscle injury (n = 3). (**e**) Quantification of monocytes in peripheral blood from Ctrl and cKO mice left untreated (n = 5) and on day 2 after muscle injury (n = 3). Data are expressed as the means ± SEM. * *p* < 0.05 with unpaired two-tailed Student’s *t*-test (**a**, **b**, **d**, **e**) or two-way ANOVA with Bonferroni’s multiple comparisons test (**c**).

Within the bone marrow, neutrophils undergo differentiation from granulocyte-monocyte progenitors, after which the fully differentiated neutrophils and are eventually released into the systemic circulation^34^.While myeloid *Arntl* deficiency is known to abolish rhythmic migration during systemic inflammation^35^, the involvement of *Arntl* in neutrophils mobilization from bone marrow in response to acute skeletal muscle inflammation is not clear from previous reports. In the present study, flow cytometry revealed that neutrophil numbers in bone marrow and the peripheral blood did not differ significantly between cKO and Ctrl mice (Fig. S5a–c). Neutrophils are recruited to sites of injury through interactions between chemokines (C-X-C motif) ligand 1 (*Cxcl1*)^36^ and ligand 2 (*Cxcl2*)^37^ and their receptor, chemokine (C-X-C motif) receptor 2 (*Cxcr2*). When we analyzed whole TA muscle tissue to test reduced expression of any of these mediators could account for the reduced neutrophil recruitment seen cKO mice, we detected no significant difference in *Cxcl1* or *Cxcl2* expression between cKO and Ctrl muscle (Fig. S6a, *p* > 0.05). Using neutrophils isolated from bone marrow, we also tested whether *Arntl* deletion affected neutrophil migration by altering expression of *Cxcr2*^38^. We found that *Cxcr2* expression of was significantly lower in cKO than Ctrl neutrophils (Fig. 4a,b), which would be expected to decrease neutrophil recruitment to the chemokines^39,40^. To assess changes in neutrophil migration due to *Arntl* deficiency, we performed chemotaxis assays in vitro, which confirmed that chemotaxis to CXCL2 was reduced in cKO neutrophils (Fig. 4c). It thus appears that loss of *Arntl* suppresses expression of *Cxcr2* in neutrophils and reduces their migration to the site of injury.

### *Arntl* is needed to increase the number of circulatory monocytes

Monocytes are produced in the bone marrow from hematopoietic stem cells through multiple processes of development and commitment as follows: common myeloid progenitors, granulocyte and macrophage progenitors, macrophage-dendric cell progenitors, common monocyte progenitors and mature monocytes^41^. Expression of *Lyz2* is also observed in macrophage-dendritic cell progenitors as well as common monocyte progenitors^42^. This suggests, *Lyz2-Cre-dependent Arntl* deficiency could potentially inhibit normal development from macrophage-dendritic cell and common monocyte progenitors to mature monocytes in the bone marrow. Our flow cytometric analysis showed that, as previously reported^3,6^, numbers of monocytes in the bone marrow and blood were increased after muscle injury in Ctrl mice (Fig. 4d,e). On the other hand, the numbers of Ly6C^hi^ monocytes in the bone marrow and blood were lower in cKO than Ctrl mice both before and 2 days after injury (Fig. 4d,e).

Within injured tissue, chemokine (C–C motif) ligand 2 (*Ccl2*)^43^ and chemokine (C-X3-C motif) ligand 1 (*Cx3cl1*)^44^ are important mediators of monocyte/macrophage recruitment and infiltration into injury sites^3,45^. Levels of their mRNA expression were similar between cKO and Ctrl muscles (Fig. S6b, *p* > 0.05). Likewise, monocyte^46^ migration assays performed with monocytes isolated from bone marrow revealed no significant difference in the expression levels of *Ccr2* and *Cx3cr1* between Ctrl and cKO monocytes (Fig. S5d). This suggests reductions in the numbers of bone marrow and circulating monocytes, not changes in the expression of chemokines or their receptors, is the primary mechanism contributing to the reduced recruitment of monocytes to regenerating muscle in cKO mice.

## Discussion

Time-dependent infiltration of myeloid cells plays a critical role in skeletal muscle regeneration. To the best of our knowledge, the present study is the first investigation to examine skeletal muscle regeneration in myeloid-cell-specific *Arntl*-deficient mice. Our findings show that *Arntl* expressed in myeloid cells is essential for normal skeletal muscle regeneration and that *Arntl* knockout in myeloid cells (1) impairs infiltration by neutrophils and monocytes into the muscle injury site; (2) suppresses expression of *Cxcr2* in neutrophils in the bone marrow, thereby reducing their chemotactic migration; and (3) leads to a reduction in the numbers of Ly6C^hi^ monocytes in the bone marrow. These results suggest that *Arntl* in myeloid cells plays an essential role in neutrophil and monocyte infiltration for skeletal muscle regeneration.

*Cxcr2* is a major chemokine receptor expressed by neutrophils^38^. Reduced neutrophil recruitment in *Cxcr2*-deficient models has been linked to delays in bacterial clearance and tissue repair^47,48^. We detected decreased numbers of infiltrating cKO neutrophils at sites of muscle injury, decreased expression of *Cxcr2* in neutrophils isolated from the bone marrow of cKO mice, and decreased chemotaxis of cKO neutrophils. These results are consistent with the reduction in neutrophil infiltration seen in *Cxcr2*-deficient mice and suggest that decreased expression of *Cxcr2* in cKO neutrophils contributed to the reduction in neutrophil infiltration into damaged muscle tissue we observed in cKO mice.

*Ccr2* and *Cx3cr1* are major chemokine receptors mediating tissue infiltration by monocytes/macrophages^46^. We detected no significant difference in levels of *Ccr2* and *Cx3cr1* expression in bone marrow monocytes between cKO and Ctrl mice. This may indicate that factors other than monocyte *Ccr2* and *Cx3cr1* expression contribute to the suppression of monocyte infiltration into skeletal muscle after injury in myeloid *Arntl*-deficient mice. The numbers of monocytes in bone marrow increases after muscle injury^6,49^, and a lack of bone marrow monocytes leads to a decrease in monocytes infiltrating muscle tissue^49^. In cKO mice, we observed a decrease in the numbers of monocytes in the bone marrow. It follows then that this reduction in the numbers of bone marrow monocytes likely led to reduced recruitment of monocytes to muscle tissue in cKO mice. Monocytes are produced in bone marrow from hematopoietic stem cells through multiple processes of development and commitment^41^. Expression of *Lyz2* is also observed in macrophage-dendritic cell and common monocyte progenitors, which are middle stages of monocyte differentiation^42^. *Lyz2-Cre-dependent Arntl* deficiency may therefore inhibit normal monocyte production in the bone marrow. However, the present study provides no direct evidence of the critically affected process steps.

Depleting circulating monocytes in mouse models using clodronate^50,51^ or CD11b diphtheria toxin receptor^52,53^ has been shown to reduce inflammatory cell infiltration during the early stages of muscle regeneration, which causes necrotic fibers to persist and impairs muscle regeneration^8,49,54^. In the present study, cKO mice exhibited decreased infiltration by monocytes into injured muscle on day 2 after injury and the persistent presence of necrotic fibers on day 4 after injury. These results are consistent with muscle regeneration in a circulating monocyte depletion model and suggest that the impaired muscle regeneration seen in cKO mice reflects the reduction in monocyte infiltration. In the present study, however, *Arntl* deletion in myeloid cells did not lead to retention of necrotic fibers to the late stage after injury and did not significantly delay in muscle regeneration, though it decreased the number of circulating monocytes. In contrast to a general monocyte depletion model, in which circulating monocytes are reduced by 80–90%^8,54^, *Arntl* deficiency induced only a 20% reduction in the circulating monocyte in the present study (Fig. 4d). That numbers of circulating monocytes were not dramatically reduced may account the lack of a dramatic delay in cKO muscle regeneration.

Previous studies demonstrated that *Arntl* deletion from macrophages increases production of pro-inflammatory cytokines via TLR4 activation^11–14^. Our earlier report using cultured macrophages confirmed that loss of *Arntl* causes a prolonged inflammatory response in macrophages and delays its convergence^15^. Similarly, we observed that in vitro and in vivo *Il6* expression was increased in our cKO macrophages. Many of the cytokines expressed in cultured macrophages, including IL6, are also reportedly expressed in macrophages in muscle tissue during regeneration and are involved in regenerative regulation^8,10^. These findings suggest there are similarities between cultured *Arntl*-cKO macrophages and regenerating skeletal muscle macrophages. IL6 acts mainly on the activation and proliferation of muscle stem cells during muscle regeneration^33,55^. The result of experiment in which muscle stem cell cultured in conditioned medium from Ctrl and cKO peritoneal macrophages confirmed that the *Arntl*-cKO macrophage-conditioned medium decreases muscle differentiation (Fig. S4). These results suggest that, apart from suppressing myeloid cell infiltration, the lack of convergence of macrophage-derived IL6 production may inhibit eventual skeletal muscle regeneration.

Because granulocytes, monocytes, and macrophages are affected in the *Lyz2cre*-deficient model, it is difficult to determine which cell type is primarily responsible for the observed inflammatory phenotype^25,56^. Neutrophils are known to contribute to the phagocytic removal of tissue debris and recruitment of monocytes^57–59^. Whether they are necessary for monocyte infiltration after muscle injury remains controversial, however^60,61^. The present study does not clarify whether the observed reduction in monocyte infiltration into skeletal muscle reflects a reduction in neutrophil infiltration. More detailed studies of specific cell types will be required to assess the contribution of *Arntl* to the recruitment of each myeloid cell to damaged skeletal muscle.

In conclusion, we have discovered that *Arntl* plays an essential role in skeletal muscle tissue regeneration after injury by regulating the appropriate timing of the gradual infiltration of neutrophils and monocytes into the injured muscle early during regeneration. The results of this study, in which the peripheral clocks of myeloid cells failed to function appropriately and inhibited muscle regeneration suggests that this mechanism may also contribute to the muscle atrophy that occurs in the space where circadian rhythms is disrupted.

## Methods

All experimental procedures were conducted according to the protocol approved by the President of Nippon Medical School after the reviewed by the Nippon Medical School Animal Care and Use Committee (Approval No. 30-027) and adhered to the relevant guidelines and regulations concerning the management and handling of experimental animals. This study is reported in accordance with the ARRIVE guidelines (https//arriveguidelines.org).

### Animals

Eight- to 12-week-old mice, housed at 22ºC under a 12-h light:dark cycle and fed ad libitum, were used in these experiments. *Arntl *^*flox/flox*^ mice were generated on a C57BL/6 background as described previously^24^. To generate *Lyz2Cre*^+*/−*^* Arntl *^*flox/flox*^ mice, *Arntl *^*flox/flox*^ mice were crossed with *Lyz2Cre* mice (*B6.129P2-Lyz2tm1(cre)Ifo/*J).

### Muscle regeneration

To induce muscle injury, the mice anesthetized with isoflurane (1.5–2%) (Pfizer, NY, USA) and 100 μl of 10 mM CTX (Sigma-Aldrich, St. Louis, MO, USA) were injected into the TA muscle of anesthetized mice using a 29 G syringe at 6 pm (ZT 10). TA muscles were then collected at 8 am (ZT 0) 1, 2, 4, or 7 days after CTX injection, fixed by immersion in Tissue-Tek Ufix (Sakura finetek, Tokyo, Japan), embedded in paraffin blocks, and cut into 10-μm-thick sections. The sections were then deparaffinized, rehydrated, stained with hematoxylin for 3 min, washed with running water for 15 min, stained in eosin for 15 min, and quickly washed in a 70, 80, 90, 95, and 100% ethanol series before finally washing twice in xylene. Images of hematoxylin/eosin-stained sections were then acquired using a microscope (BZ-X810, Keyence, Osaka, Japan) and analyzed to assess the minor fiber axis and to quantify regenerating and necrotic fibers. The numbers of regenerating fibers that had centrally located myonuclei were counted, and the minor axis was measured in 2000–3000 regenerating fibers in each mouse. Necrotic fibers were identified as round myofibers lacking the centrally-located nuclei prevalent in regenerated myofibers^62,63^. The necrotic fiber area was measured as the sum of the areas of the necrotic fibers in each section.

To obtain immunofluorescence images, TA muscles were isolated 7 days after CTX injection, immediately frozen in cooled isopentane in liquid nitrogen, and stored at − 80 °C. For immunostaining, the muscles were cut into 10-μm-thick cryosections, which were stained for myosin-heavy polypeptide 3 (MYH3, clone F1.652, Santa Cruz, Dallas, Texas, USA) and with 4',6-diamidino-2-phenylindole dihydrochloride (DAPI). Images of MYH3- and DAPI-stained sections were acquired using a confocal microscope (SP5, Leica, Camera AG, Wetzlar, Germany).

### Preparation of cells for flow cytometry

Isolated TA muscles were homogenized and digested with collagenase II solution (2 µg/ml, Worthington Biochemical, Lakewood, NJ, USA) for 1 h at 37 °C in a shaker. After first suspending bone marrow cells and blood samples in red blood cell lysis buffer (Invitrogen, Carlsbad, CA, USA), the cell suspensions were passed through 100-μm strainers, centrifuged, aspirated, resuspended, and passed through 40-μm strainers. Pelleted cells were then resuspended in PBS with 2% FBS for antibody staining and analysis. The following antibodies directed against mouse antigens were used: BV421-conjugated CD45 (clone 30-F11, BioLegend, San Diego, CA, USA), PECy7-conjugated CD11b (clone M1/70, BD Biosciences, Franklin Lakes, NJ, USA), APCCy7-conjugated Ly6G (clone 1A8, BioLegend), APC-conjugated Ly6C (clone HK1.4, BioLegend), PE-conjugated F4/80 (clone T45-2342, BD Biosciences), FITC-conjugated Siglec-F (clone S17007L, BioLegend) and PE-conjugated CXCR2 (clone SA044G4, BioLegend). Data were acquired with a FACSAriaIII (BD, Franklin Lakes, NJ, USA) and LSRFortessa (BD) and analyzed using FlowJo v10 (Treestar, San Francisco, CA, USA). In addition, numbers of live cells were counted. The absolute value of each cell count was calculated by multiplying the percentage of the total cells counted that were live by the number of live cells. Cell counts for muscle tissue were expressed per unit tissue weight, peripheral blood per unit blood volume, and bone marrow per tibia.

### Migration assays

Neutrophil migration was assessed using 24-well microchambers and polycarbonate filters (5 μm pore size) (Corning, NY, USA) as described previously^40,64^. In brief, CD45^+^ CD11b^+^ Ly6G^+^ neutrophils sorted from mouse bone marrow cells were placed in the upper wells (1 × 10^5^ cells/well) of Transwell chambers, and 600 µL of RPMI 1640 medium with or without 100 ng/ml CXCL2 (BioLegend) were added to the lower wells. For migration assays, cells were incubated for 60 min at 37 °C. After the incubation, cells that migrated to the bottom part of the membrane and the lower wells was stained with Hoechst 33,342. The numbers of cells per field were counted in 10 randomly selected visual fields under a microscope (BZ-X810, Keyence, Osaka, Japan), and the mean estimate for individual samples was calculated.

### Quantitative RT-PCR

Total RNA was isolated from homogenized TA muscles or cultured cells using ISOGEN (Nippon Gene, Tokyo, Japan). The RNA was isolated using the phenol–chloroform extraction and isopropanol precipitation protocol according to the manufacturer’s instructions. Total RNA was extracted from sorted cells using a RNeasy Mini Kit (Qiagen, Valencia, CA, USA). Complementary DNA (cDNA) was synthesized using ReverTra Ace qPCR RT Master Mix with genomic DNA Remover (TOYOBO CO., LTD., Osaka, Japan). cDNA was analyzed with real-time PCR in a QuantStudio 5 Real-time PCR system (Applied Biosystems, Foster City, CA, USA) using PowerUp SYBR Green Master Mix (Applied Biosystems). The primers used for qPCR are listed in Table 1. Glyceraldehyde-3-phosphate dehydrogenase (*Gapdh*) expression was used as an internal control.

**Table 1:** The primers used in this study.

	Forward	Reverse
*Gapdh*	AATGTGTCCGTCGTGGATCT	CATCGAAGGTGGAAGAGTGG
*Arntl*	TGCAGAACACCAAGGAAGGAT	GTTCATTTTGTCCCGACGCC
*Ckm*	CTGACCCCTGACCTCTACAAT	CATGGCGGTCCTGGATGAT
*Myom2*	AAAAGACACAAGCACTTTGACCA	TGGGAGGATGACTGGGTGG
*Il6*	ATGGATGCTACCAAACTGGAT	TGAAGGACTCTGGCTTTGTCT
*Cxcl1*	CTGGGATTCACCTCAAGAACATC	CAGGGTCAAGGCAAGCCTC
*Cxcl2*	CGCTGTCAATGCCTGAAG	GGCGTCACACTCAAGCTCT
*Cxcr2*	TCTGGCATGCCCTCTATTCTG	AAGGTAACCTCCTTCACGTAT
*Ccl2*	TTAAAAACCTGGATCGGAACCAA	GCATTAGCTTCAGATTTACGGGT
*Ccr2*	ACCTGTAAATGCCATGCAAGT	TGTCTTCCATTTCCTTTGATTTG
*Cx3cl1*	ACGAAATGCGAAATCATGTGC	CTGTGTCGTCTCCAGGACAA
*Cx3cr1*	AGTTCCCTTCCCATCTGCTC	AATGTCGCCCAAATAACAGG

### Bone marrow-derived macrophages and peritoneal macrophages

To culture bone marrow-derived macrophages, bone marrow was collected from mice by perfusing the medullary cavities of the femur and tibia and suspended in red blood cell lysis buffer. The cells were then cultured in RPMI-1640 medium containing 10% FBS and 45 ng/ml M-CSF (BioLegend) in 15 cm Petri dishes. The medium was changed after 3 days, and the cells were collected on day 5 of differentiation. To prepare peritoneal macrophages, cells were collected from the peritoneum of mice in exudate after peritoneal lavage. The collected cells were cultured for 1 h, then washed with PBS to remove nonadherent cells. The adherent cells were cultured as macrophages overnight^65^. Some of the macrophages were stimulated for 6 h with the specific TLR4 agonist Kdo2-lipid A (KLA, 100 ng/ml, Sigma-Aldrich, St. Louis, MO, USA)^66^. Untreated or stimulated macrophages were subsequently harvested for RNA extraction.

### IL-6 ELISA

Cultured peritoneal macrophages (1 × 10^5^ cells/500 µl/well) were stimulated for 24 h with 100 ng/ml KLA, after which the cells were collected and centrifuged for 10 min at 10,000 r.p.m. The resulting supernatant was assayed for IL-6 using a mouse IL-6 ELISA kit (R&D, Minneapolis, MN, USA) according to the manufacturer’s instructions. Optical density was measured using an EnSpire Multimode Plate Reader (PerkinElmer, Waltham, MA, USA).

### Myotube differentiation from muscle stem cells

Mouse primary muscle stem cells were isolated from hindlimb muscles from C57BL/6 J wild-type mice. After excess fat, connective tissue and tendons were removed, hindlimb muscles were minced and digested in collagenase II solution (2 µg/ml, Worthington Biochemical, Lakewood, NJ, USA) for 1 h at 37 °C. Cells were stained with PECy7-conjugated CD31 (clone 390, BD Pharmingen), CD45 (clone 30-F11, BD Pharmingen), and Ly6A/E (clone D7, BD Pharmingen) as well as FITC-conjugated CD106 (clone 429, BioLegend) antibodies for 30 min on ice and resuspended in PBS with 2% FBS. Muscle stem cells were then isolated using FACSAriaIII (BD). Satellite cells were cultured in GlutaMax DMEM (Life Technologies, Carlsbad, CA) supplemented with 20% FBS, 10 ng/mL basic fibroblast growth factor (Cell Signaling Technology, Beverly, MA, USA), and 0.2 µg/cm^2^ iMatrix-511 silk (Takara bio, San Jose, CA, USA) at 37 °C. Myogenic differentiation was induced in GlutaMax DMEM supplemented with 5% horse serum on a Matrigel-coated plate at 37 °C.

For preparation of macrophage-conditioned medium, peritoneal macrophages were cultured first in RPMI-1640 medium containing 10% FBS at 37 °C overnight and then RPMI-1640 without FBS for an additional 24 h. Muscle stem cells were cultured for 2 days in new conditioned differentiation medium composed of macrophage-conditioned and differentiation medium as described previously^67^.

For myotube differentiation assays, myotubes were fixed in 4% paraformaldehyde solution and then inoculated with anti-skeletal muscle myosin antibody (MYH, clone F59, Santa Cruz) and stained with Hoechst 33342. Images of myotubes were acquired from randomly selected visual fields under a microscope (BZ-X810, Keyence). Ratio of nuclei within myotubes in individual samples were counted as a fusion index^68^.

### Immunoblotting

Protein was extracted from cells using RIPA buffer and quantified using a BCA Protein assay kit (Pierce, Rockford, IL) following the manufacturer’s protocol. Sample were then mixed with SDS loading buffer, electrophoresed on 10% (vol/vol) acrylamide gels, transferred onto PVDF membranes, and blocked with 5% milk for 1 h before incubation with a primary antibody. The antibodies used for western blotting were rabbit anti-BMAL1 (ab93806, Abcam, Cambridge, UK) and anti-β-tubulin (clone 10G10, Wako, Osaka, Japan). The blots were then developed using ECL Prime detection reagent (GE, Waukesha, WI, USA). Raw images are in Fig. S8.

### Statistical analysis

Data are presented as means ± SEM, except where otherwise indicated. Sample sizes were not based on power calculations. Statistical significance is determined using the two-tailed Student’s *t*-test. Two-way ANOVA with post-hoc Bonferroni’s multiple comparison test was used for experiments involving two factors, except where otherwise indicated. Significance values are indicated as *p < 0.05. All statistical analyses were performed using Prism 9 (GraphPad, San Diego, CA, USA).

## Supplementary Information

Supplementary Information.

## Data Availability

The datasets generated and analysed during the current study are available in this published article and its supplementary information files.
