# Supplementary material for: Arntl deficiency in myeloid cells reduces neutrophil recruitment and delays skeletal muscle repair
Source: Sci Rep. 2023 Apr 25;13:6747. doi: 10.1038/s41598-023-33830-8 (PMC10130093; doi:10.1038/s41598-023-33830-8)
Supplement: Supplementary file 1 — Supplementary Information. [file 41598_2023_33830_MOESM1_ESM.pdf]

Arntl deficiency in myeloid cells reduces neutrophil recruitment and delays skeletal muscle repair

Aiko Watanabe[1,2], Hiroyuki Koike[1,*], Naoki Kumagami[1,2], Shigeki Shimba[3], Ichiro Manabe[4], Yumiko Oishi[1,*]

1. Department of Biochemistry & Molecular Biology, Nippon Medical School, 1-1-5 Sendagi, Bunkyo-ku, Tokyo 113-8602, Japan.
2. Department of Molecular Cell Biology, Graduate School of Medical and Dental Sciences, Tokyo Medical and Dental University, 1-5-45, Yushima, Bunkyo-ku, Tokyo 113-8510, Japan.
3. Department of Health Science, School of Pharmacy, Nihon University, 7-7-1 Narashinodai, Funabashi, Chiba 274-8555, Japan
4. Department of Systems Medicine, Graduate School of Medicine, Chiba University, 1-8-1 Inohana, Chuo-ku, Chiba-shi, Chiba 260-8670, Japan.

*. To whom correspondence should be addressed Hiroyuki Koike, E-mail: h-koike@nms.ac.jp, and Yumiko Oishi, yuooishi@gmail.com

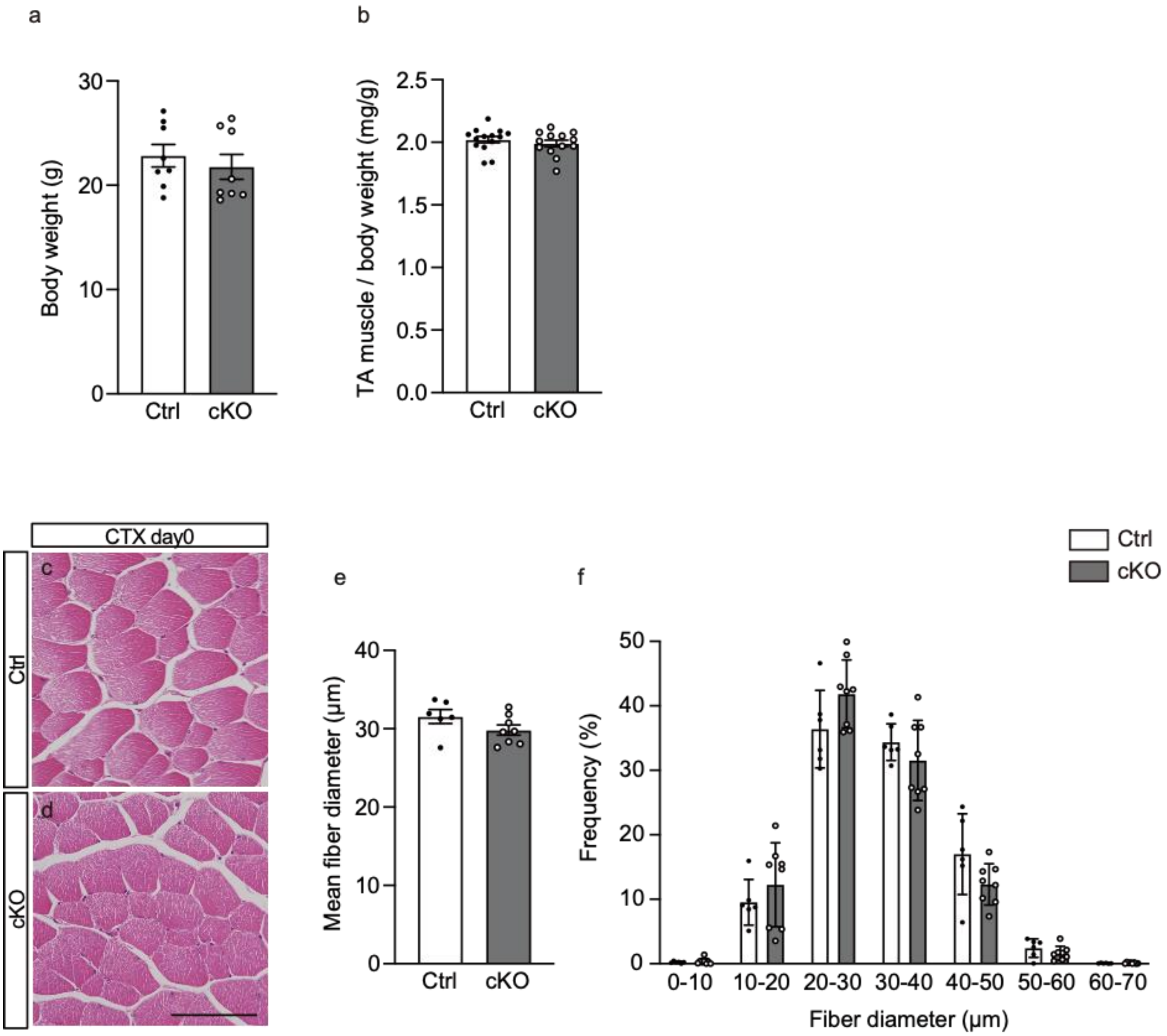

Supplementary Figure S1. ***Arntl*** **deletion from myeloid cells does not significantly change skeletal muscle phenotype pre-injury.**

a; Bodyweight of untreated Ctrl (n = 8) and cKO (n = 8) mice. b; TA muscle mass per body weight of untreated Ctrl (n = 14) and cKO (n = 13) muscles. c,d; Hematoxylin/eosin staining of untreated Ctrl and cKO TA muscles. Representative images from n = 4 Ctrl and cKO mice. e; Mean fiber diameter of untreated Ctrl (n = 6) and cKO (n = 8) TA muscles. f; Fiber diameter distributions of Ctrl (n = 6) and cKO (n = 8) untreated TA muscles. Data are expressed as the

means ± SEM. * $p < 0.05$ with unpaired two-tailed Student's *t*-test for (a, b, e) or two-way ANOVA with Bonferroni's multiple comparisons test (f). Scale bar = 100 μm.

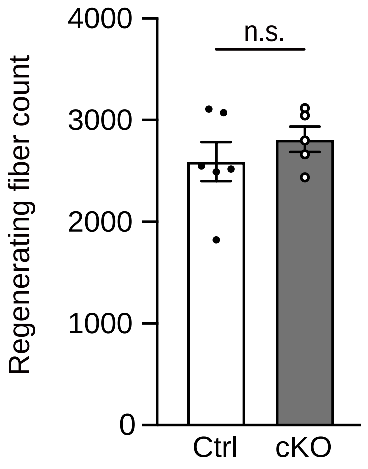

Supplementary Figure S2. *Arntl* deletion from myeloid cells does not significantly change the number of regenerating fibers.

The number of regenerating fibers in Ctrl (n = 6) and cKO (n = 5) TA muscles on day 7 post-injury. Data are expressed as the means ± SEM. not significant (n.s.); $p > 0.05$ with unpaired two-tailed Student's *t*-test.

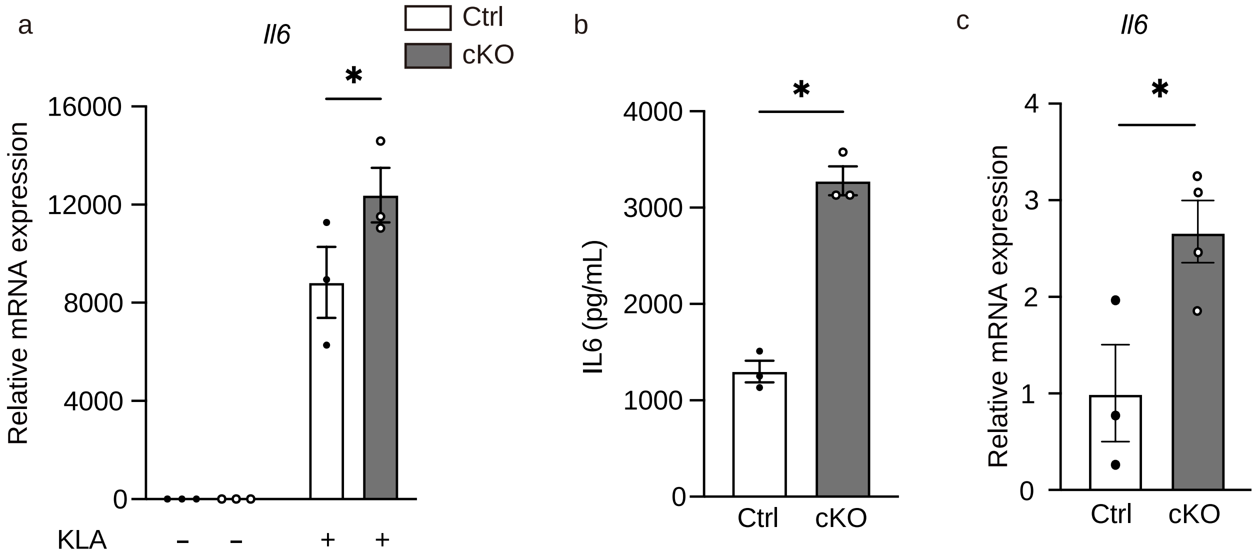

Supplementary Figure S3. ***Arntl*** **deletion from macrophages increases the expression of** ***Il6.***

a; RT-PCR assessment of interleukin 6 (*Il6*) expression in untreated and treated Ctrl (n = 3) and cKO (n = 3) peritoneal macrophages with Kdo2-lipid A (KLA) for 6h. b; Expression level of IL6 protein in untreated and treated Ctrl (n = 3) and cKO (n = 3) peritoneal macrophages with KLA for 24h. c; RT-PCR assessment of *Il6* expression in sorted myeloid cells ($CD45^{+}CD11b^{+}$) from Ctrl (n=3) and cKO (n=4) muscles on day 3 after injury. Data are expressed as the means ± SEM. * $p < 0.05$ with unpaired two-tailed Student's *t*-test for (b, c) or two-way ANOVA with Bonferroni's multiple comparisons test for (a).

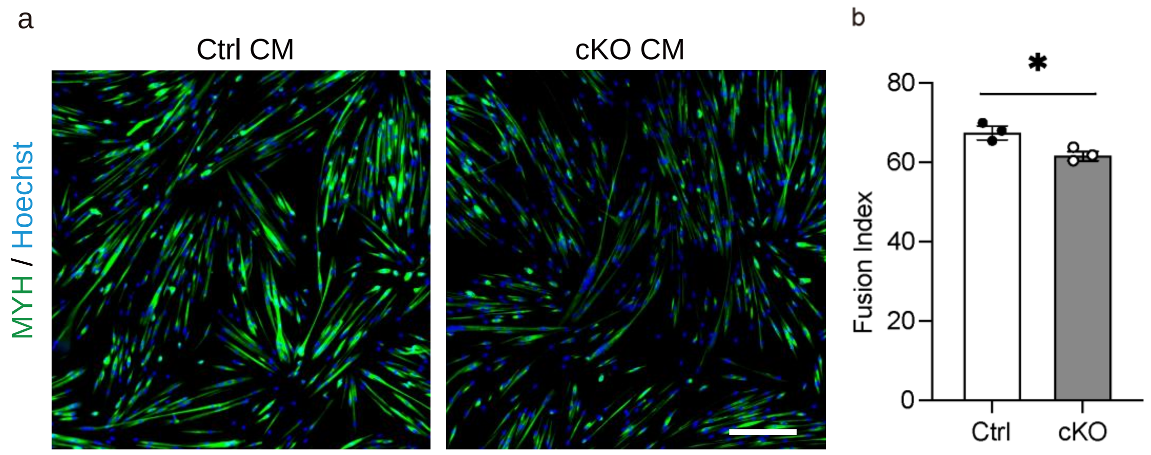

Supplementary Figure S4. Culture medium conditioned by *Arntl*-depleted macrophages significantly inhibited differentiation of muscle stem cells.

a; Immunostaining of skeletal muscle myosin (green) and Hoechst staining of nuclei (blue) from wild-type skeletal muscle stem cells cultured in conditioned medium (CM) from Ctrl and cKO peritoneal macrophages. Representative images from n = 3 Ctrl and cKO wells. b; Quantification of the fusion index from the image in (a). Ctrl (n = 3) and cKO (n = 3) wells. Data are expressed as the mean ± SEM. * $p < 0.05$ with unpaired two-tailed Student's *t*-test. Scale bar = 200 µm.

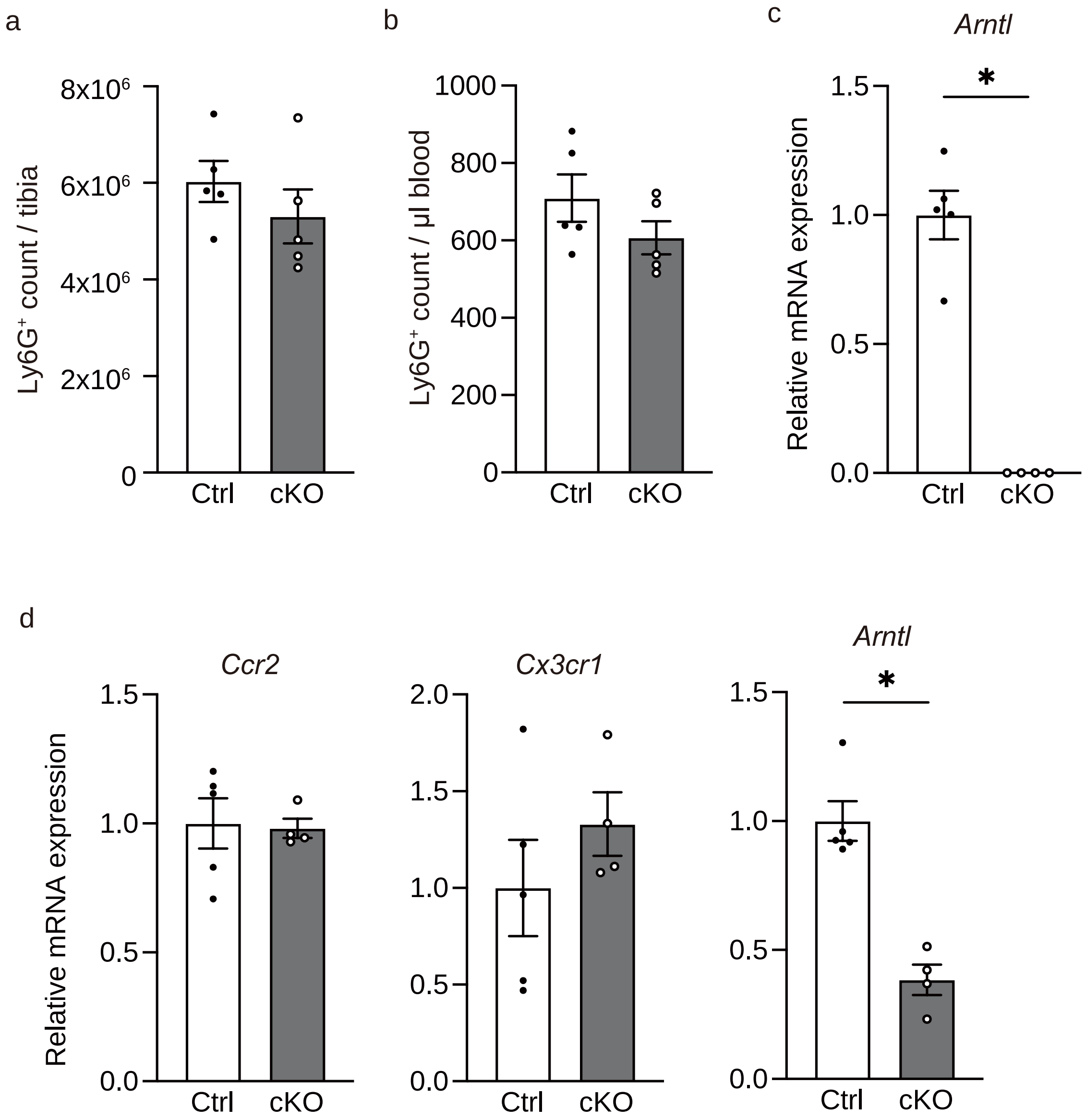

Supplementary Figure S5. *Arntl* deletion from myeloid cells does not significantly change the number of circulating neutrophils and the migration-related gene expressions in monocytes.

a; Quantification of neutrophils ($CD45^+$ $CD11b^+$ $Ly6G^+$) in bone marrow cells from Ctrl (n = 5) and cKO (n = 5) mice left untreated. b; Quantification of neutrophils ($CD45^+$ $CD11b^+$ $Ly6G^+$) in blood from Ctrl (n = 5) and cKO (n = 5) mice left untreated. c; RT-PCR assessment of *Arntl* expression in neutrophils ($CD45^+$ $CD11b^+$ $Ly6G^+$) sorted from untreated bone marrow cells from

Ctrl (n = 5) and cKO (n = 4) mice. d; RT-PCR assessment of chemokine (C-C motif) receptor 2 (*Ccr2*), chemokine (C-X3-C motif) receptor 1 (*Cx3cr1*), and *Arntl* expression in monocyte ($CD45^{+}$ $CD11b^{+}$ $Ly6G^{-}$ Siglec-F^{-} $Ly6C^{hi}$) sorted from bone marrow cells from Ctrl (n = 5) and cKO (n = 4) mice left untreated. Data are expressed as the means ± SEM. * $p < 0.05$ with unpaired two-tailed Student's *t*-test.

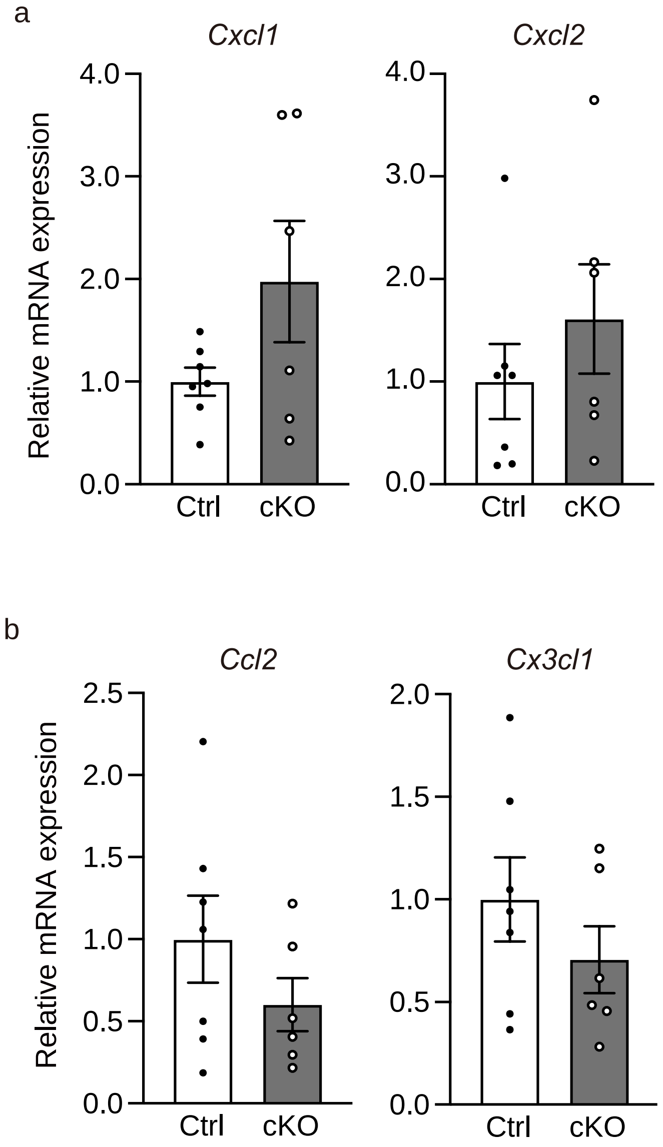

Supplementary Figure S6. *Arntl* deletion from myeloid cells does not significantly change the expression of chemokines related to neutrophil and monocyte migration in skeletal muscle.

a; RT-PCR assessment of chemokine (C-X-C motif) ligand 1 (*Cxcl1*) and chemokine (C-X-C motif) ligand 2 (*Cxcl2*) expressions in the TA muscles from Ctrl (n = 7) and cKO (n = 6) muscles on day one after muscle injury. b; RT-PCR assessment of (C-C motif) receptor 2 (*Ccl2*) and chemokine (C-X3-C motif) ligand 1 (*Cx3cl1*) expressions in the TA muscles from Ctrl (n = 7)

and cKO (n = 6) muscles on day 2 after muscle injury. Data are expressed as the means ± SEM.

* $p < 0.05$ with unpaired two-tailed Student's *t*-test.

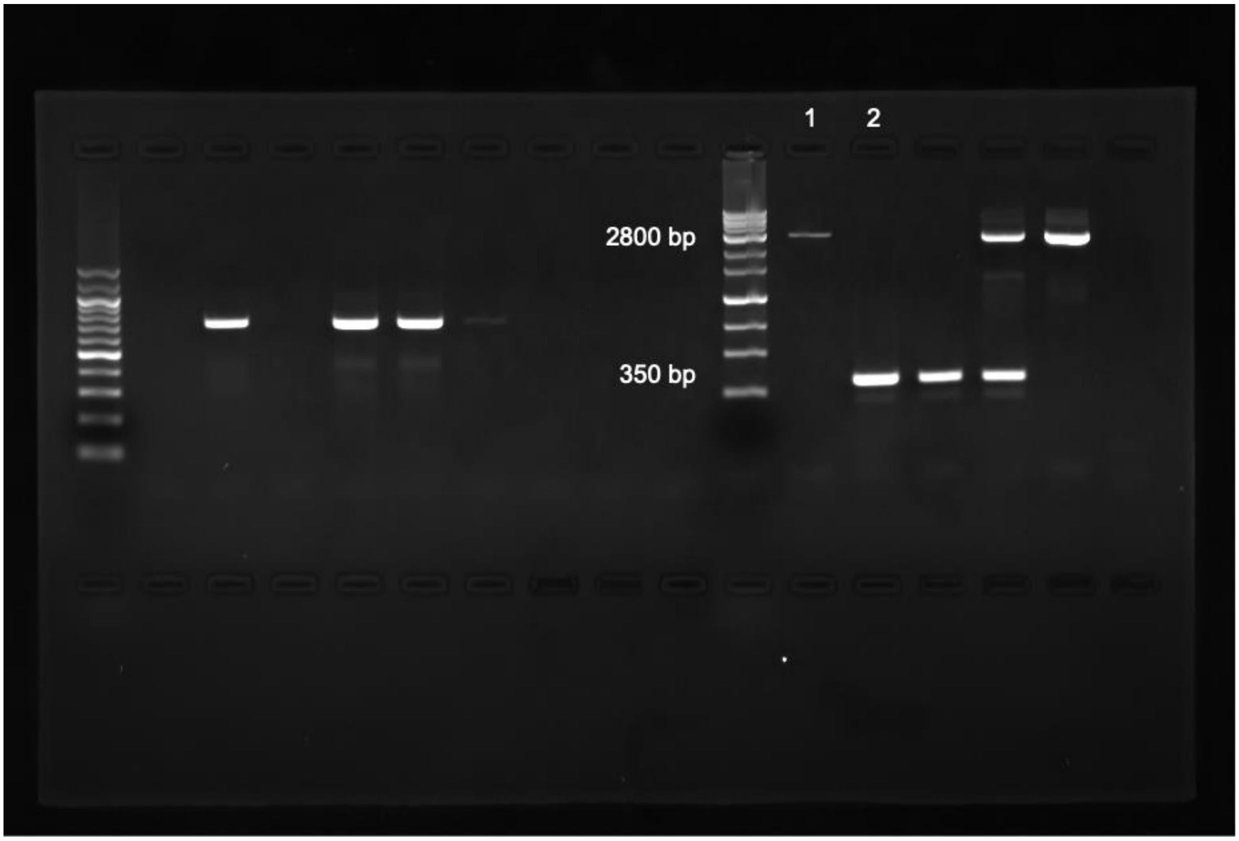

1 : *Arntl*$^{flox/flox}$
2 : *Lyz2Cre*$^{+/-}$ *Arntl*$^{flox/flox}$

Supplementary Figure S7. PCR confirms the *Arntl* deletion in bone marrow-derived macrophages (BMDMs) from myeloid cell-specific *Arntl* knockout mice.

The image of the original blots of the experiment corresponds to Figure 1b.

a

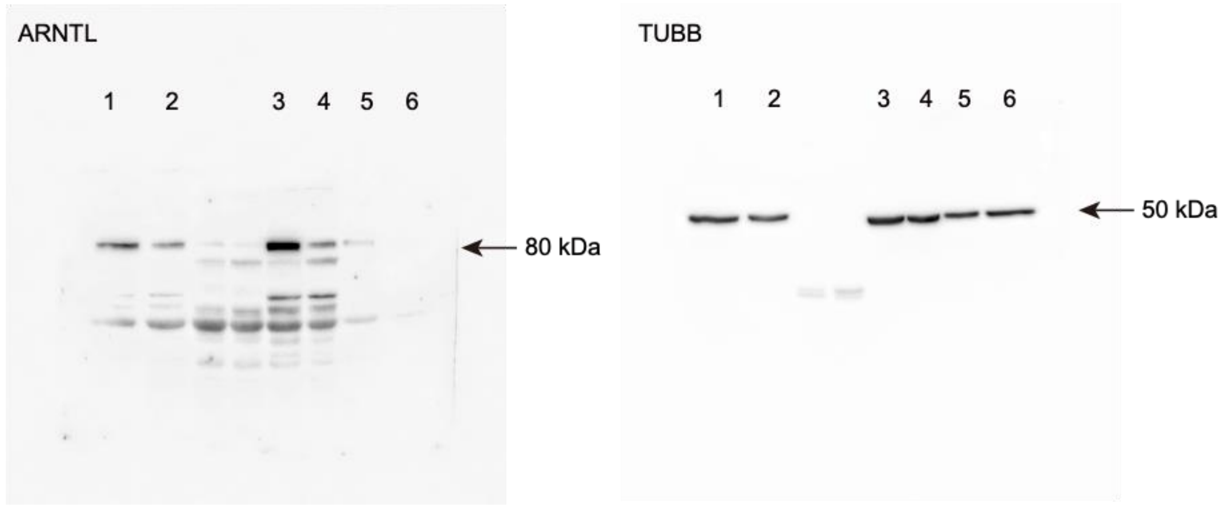

1 : BMDM *Arntl*$^{flox/flox}$

2 : BMDM *Lyz2Cre*$^{+/-}$ *Arntl*$^{flox/flox}$

3 : BMDM *Arntl*$^{flox/flox}$

4 : BMDM *Lyz2Cre*$^{+/-}$ *Arntl*$^{flox/flox}$

5 : PEC *Arntl*$^{flox/flox}$

6 : PEC *Lyz2Cre*$^{+/-}$ *Arntl*$^{flox/flox}$

b

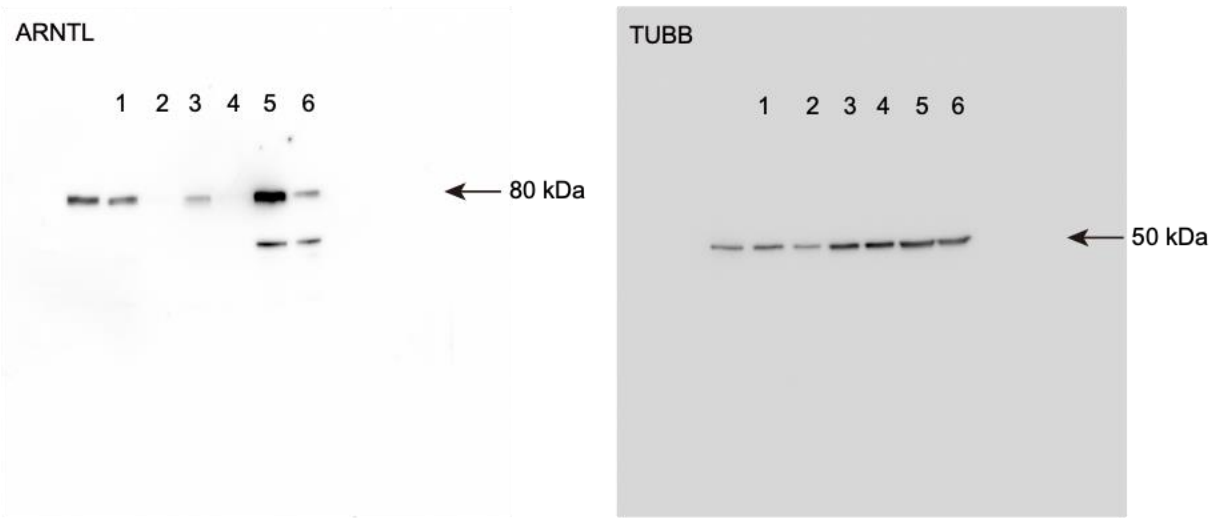

1 : PEC *Arntl*$^{flox/flox}$

2 : PEC *Lyz2Cre*$^{+/-}$ *Arntl*$^{flox/flox}$

3 : PEC *Arntl*$^{flox/flox}$

4 : PEC *Lyz2Cre*$^{+/-}$ *Arntl*$^{flox/flox}$

5 : BMDM *Arntl*$^{flox/flox}$

6 : BMDM *Lyz2Cre*$^{+/-}$ *Arntl*$^{flox/flox}$

Supplementary Figure S8. Western blots confirm the *Arntl* deletion in BMDM and peritoneal exudate cells (PECs) from myeloid cell-specific *Arntl* knockout mice.

a; Image of the original blots of the first experiment corresponds to Figure 1c. The membrane hybridized with ARNTL antibody was reprobed with tubulin beta (TUBB) antibody. b; Image of the original blots of the second experiment corresponds to Figure 1c.
